# Dynamic analysis of peripheral blood TCR β-chain CDR3 repertoire in occupational medicamentosa-like dermatitis due to trichloroethylene

**DOI:** 10.1038/s41598-021-89431-w

**Published:** 2021-05-11

**Authors:** Dafeng Lin, Dianpeng Wang, Peimao Li, Xiangli Yang, Wei Liu, Lu Huang, Zhimin Zhang, Yanfang Zhang, Wen Zhang, Naixing Zhang, Ming Zhang, Xianqing Huang

**Affiliations:** 1 Medical Laboratory, Shenzhen Prevention and Treatment Center for Occupational Diseases, 2019 Buxin Rd., Luohu district, Shenzhen, 518020 China; 2 Key Laboratory of Modern Toxicology of Shenzhen, Medical Key Laboratory of Guangdong Province, Medical Key Laboratory of Health Toxicology of Shenzhen, Shenzhen Center for Disease Control and Prevention, Shenzhen, 518055 China; 3 Fuyong Prevention and Health Care Center, Bao’an District, Shenzhen, 518103 China

**Keywords:** Adaptive immunity, VDJ recombination, Epidemiology, Genetics research, Immunological disorders, Skin diseases, Immunopathogenesis

## Abstract

Previously, we had cross-sectionally explored the characteristics of T cell receptor (TCR) repertoires from occupational medicamentosa-like dermatitis due to trichloroethylene (OMDT) patients, now we further analyzed the dynamic features of OMDT TCR repertoires. Peripheral blood TCR β-chain complementarity-determining region 3 (CDR3) genes were detected with the high throughput sequencing in 24 OMDT cases in their acute, chronic and recovery stages, respectively, and in 24 trichloroethylene-exposed healthy controls. The TCR repertoire diversity, TRBV/TRBD/TRBJ gene usage and combination, frequencies of CDR3 nucleotide (nt) and amino acid (aa) sequences in the cases in different stages and in the controls were analyzed. TRBV6-4 and TRBV7-9 frequencies significantly differed between the cases and controls (both *P* < 6.1 × 10^–4^). TRBV6-4 combination with TRBJ2-1, TRBJ2-2, TRBJ2-3, and TRBJ2-6, and TRBV7-9 combination with TRBJ2-1 were associated with the stage by OMDT severity (all *P* < 0.001). Ten CDR3-nt and 7 CDR3-aa sequences in TRBV7-9-TRBJ2-1 combination and 1 CDR3-nt and 1 CDR3-aa sequences in TRBV6-4-TRBJ2-1 combination were identified as associated with the severity of OMDT (all *P* < 0.001). We revealed further how TCR repertoires vary with the severity in the development of OMDT, and severity-related TCRs may provide important therapeutic targets for OMDT in clinical practice.

## Introduction

Trichloroethylene (TCE) is known as an environmental contaminant and an industrial toxicant mainly used as organic solvent and degreasing agent, mostly in some Asian countries like China, Japan and Korea over the last few decades^1,2^. Exposure to TCE was reported to cause a notorious occupational disease named occupational medicamentosa-like dermatitis due to trichloroethylene (OMDT). The manifestation of OMDT includes various degrees of generalized skin lesions, fever, hepatitis, and lymphadenopathy. It resembles serious drug hypersensitivities referred to as drug reaction with eosinophilia and systemic symptoms (DRESS) or drug-induced hypersensitivity syndrome (DIHS)^2^. Epidemiological studies predicted that the incidence of OMDT was about 1%^2^, but more than 7% of the patients died of secondary hepatic encephalopathy, severe infection, and multiple organ failure^3^. Survived patients usually had a very long clinical course, and the symptoms could relapse after cessation of the treatment.

The clinical features of OMDT include a latency of 2–5 weeks, no dose–response relationship, skin lesions not confined to the contact area, the relatively effective glucocorticoid therapy, and rapid recurrence due to re-exposure to trichloroethylene^2,4^. Based on the above features, OMDT is generally regarded as a T cell-mediated, Type IV hypersensitivity reaction. Other pathological evidence includes that elevated levels of T cells were identified in peripheral blood and affected skin tissues of the patients^5^. Therefore, T cells may play a pivotal role in the pathogenesis of OMDT. T cell receptor (TCR) is a membrane protein of T cell responsible for recognizing antigens and transmitting signals to downstream molecules^6^. Because TCR is the key to initiate T cell reactions, studying TCR in OMDT may help to clarify the underlying pathogenetic mechanism and provide basis for more effective prevention and treatment measures.

Each T cell is known to generally encode one single, unique TCR. To ward off a wide variety of pathogens, a vast array of differential TCRs in the human adaptive immune system constitute a TCR repertoire^7^. Most of TCRs in the repertoire are composed of an α-chain and a β-chain, and each of the chains contains a variable region (V region) which has three hypervariable regions, namely complementarity-determining region (CDR) 1, CDR2, and CDR3. The most variable is the CDR3 which directly determines the antigen-binding specificity of the TCR^7^. Rearrangement of variable (V), diversity (D) and joining (J) segments occurs in the CDR3 gene during the maturity process of the lymphocyte^7^. Notably, β-chain CDR3 is the mostly studied because β-chain is a better indicator to distinguish different T cell clones from another.

Previously, we conducted a case–control study to explore the characteristics of TCR repertoires in OMDT by the high-throughput sequencing (HTS) of the TCR β-chain CDR3^8^. In the previous, cross-sectional study, we found 5 TCR β-chain V gene (TRBV) and 1 TCR β-chain J gene (TRBJ) usage, and 48 TRBV-TRBJ combinations that might be related to OMDT. We also identified 17 CDR3 nucleotide (nt) sequences and 16 CDR3 amino acid (aa) sequences of which the corresponding T cell clones might be specific to OMDT and participate in OMDT development^8^. However, the human TCR repertoire is quite sophisticated, and it actually dynamically shifts with the disease status. One cross-sectional exploration with limited sample size is difficult to comprehensively reveal the characteristics of TCR repertoires in OMDT. Therefore, we now increased the sample size and carried out a longitudinal study to further investigate the dynamic features of TCR repertoires in the course of OMDT.

## Results

### Baseline characteristics of the study subjects and blood test indices

Characteristics of the cases at admission and of the controls are listed in Table 1. The cases had an average age of 24.0 years, comparable to that of the controls (28.0 years). Seventy percent of the cases were male, and 83.3% were Han Chinese, also comparable to the percentage of male (79.2%) and of Han (91.7%) in the controls, respectively. Smoking and drinking habits were matched between the cases and controls. The cases were exposed to a higher average trichloroethylene concentration than the controls (15.2 *vs.* 1.6 mg/m^3^, *P* = 2.7 × 10^–6^), and their average trichloroethylene exposure period was significantly shorter (31.5 *vs.* 220.5 days, *P* = 2.9 × 10^–9^).

**Table 1:** Baseline characteristics of the study subjects.

Variables	OMDT cases (*n* = 24)	Controls (*n* = 24)	*P*
Age (year, *M* (*MAD*))	24.0 (8.2)	28.0 (7.4)	0.261^a^
Sex (male, %)	17 (70.8)	19 (79.2)	0.739^b^
Race (Han, %)	20 (83.3)	22 (91.7)	0.666^c^
Smoking (yes, %)	9 (37.5)	7 (29.2)	0.760^b^
Drinking (yes, %)	1 (4.2)	2 (8.3)	1.000^c^
Trichloroethylene exposure concentration _TWA_ (mg/m^3^, *M* (*MAD*))	15.2 (8.2)	1.6 (1.2)	< 0.001^a^
Trichloroethylene exposure period (day, *M* (*MAD*))	31.5 (6.7)	220.5 (60.0)	< 0.001^a^

OMDT, occupational medicamentosa-like dermatitis due to trichloroethylene; M, median; MAD, median absolute deviation; TWA, time weighted average.

^a^Mann–Whitney U test.

^b^*χ*^2^-test.

^c^Fisher’s exact test.

The averages of blood test indices in the controls in Table 2 were all within their respective clinical references. The OMDT cases in acute stage showed significantly higher white blood cell (WBC) count, neutrophil count, alanine aminotransferase (ALT) activity, and aspartate aminotransferase (AST) activity, and lower red blood cell (RBC) count, hemoglobin concentration, total protein (TP) concentration, and albumin concentration than the controls (all *P* < 0.05); the cases in acute stage also showed higher ALT and AST activities than in chronic and recovery stages (all *P* < 0.05). The cases in chronic stage had higher WBC, lymphocyte, and neutrophil counts, ALT and AST activities, and lower RBC count, hemoglobin, TP, and albumin concentrations than the controls (all *P* < 0.05), the cases in chronic stage also had higher ALT and AST activities than in recovery stage (both *P* < 0.01). The cases in recovery stage showed lower RBC count, hemoglobin, TP, and albumin concentrations than the controls (all *P* < 0.05), while the other indices were comparable to their counterparts in the controls.

**Table 2:** Comparison of blood test indices between the groups.

Variables	OMDT cases (*n* = 24)			Controls (*n* = 24)
	Acute stage	Chronic stage	Recovery sage	
White blood cell count (× 10^9^/L, *M* (*MAD*))^a^	11.75 (5.72)*	13.74 (5.81)**^#^	7.81 (3.97)	6.92 (2.09)
Lymphocyte count (× 10^9^/L, *M* (*MAD*))^a^	2.74 (1.88)	3.24 (0.95)*	2.42 (0.90)	2.37 (0.67)
Neutrophil count (× 10^9^/L, *M* (*MAD*))^a^	7.06 (3.31)*	8.79 (2.90)**^#^	4.66 (3.23)	4.20 (1.70)
Red blood cell count (× 10^9^/L, *M* (*MAD*))^a^	4.86 (0.46)*	4.29 (0.71)**	4.53 (0.47)*	5.43 (0.48)
Hemoglobin concentration (g/L, mean (*SD*))^b^	134.61 (20.59)**	129.33 (17.82)**	133.15 (18.93)**	155.12 (16.20)
Alanine aminotransferase activity (U/L, *M* (*MAD*))^a^	503.00 (434.40)**^##$^	181.00 (171.98)**^##^	25.00 (19.27)	23.50 (14.83)
Aspartate aminotransferase activity (U/L, *M* (*MAD*))^a^	199.00 (166.05)**^##$^	60.00 (51.15)**^##^	21.00 (5.93)	23.50 (7.41)
Total protein concentration (g/L, *M* (*MAD*))^a^	59.40 (6.82)**	58.45 (5.56)**	61.05 (4.08)**	76.50 (3.71)
Albumin concentration (g/L, *M* (*MAD*))^a^	34.00 (4.60)**^#^	36.30 (3.71)**	38.25 (2.59)**	47.50 (3.71)

OMDT, occupational medicamentosa-like dermatitis due to trichloroethylene; M, median; MAD, median absolute deviation; SD, standard deviation.

^a^Mann–Whitney U test with Holm’s adjustment.

^b^ANOVA with Tukey’s HSD test.

Compared with controls, *P < 0.05, **P < 0.01; compared with cases in recovery stage, ^#^*P* < 0.05, ^##^*P* < 0.01; compared with cases in chronic stage, ^$^*P* < 0.01.

### TCR repertoire diversity

The HTS yielded an average of 16,313,850 raw reads per sample. After filtration of low quality reads and adapter contamination, we obtained an average of 16,068,894 clean reads per sample. Each sample has high clean read Q20 and Q30, indicating good quality of the sequencing data. The overview of sequence statistics is shown in Supplementary Table S1 online.

We used 6 indices to measure the TCR repertoire diversity. None of the 6 indices, including resampled diversity, extrapolate diversity, chaoE, chao1, Shannon–Wiener index, and inverse Simpson index, was found significantly different between the cases and the controls, or between different stages of the cases (see Supplementary Fig. S1 online).

### TRBV/TRBD/TRBJ usage and combination

**Figure 1:**
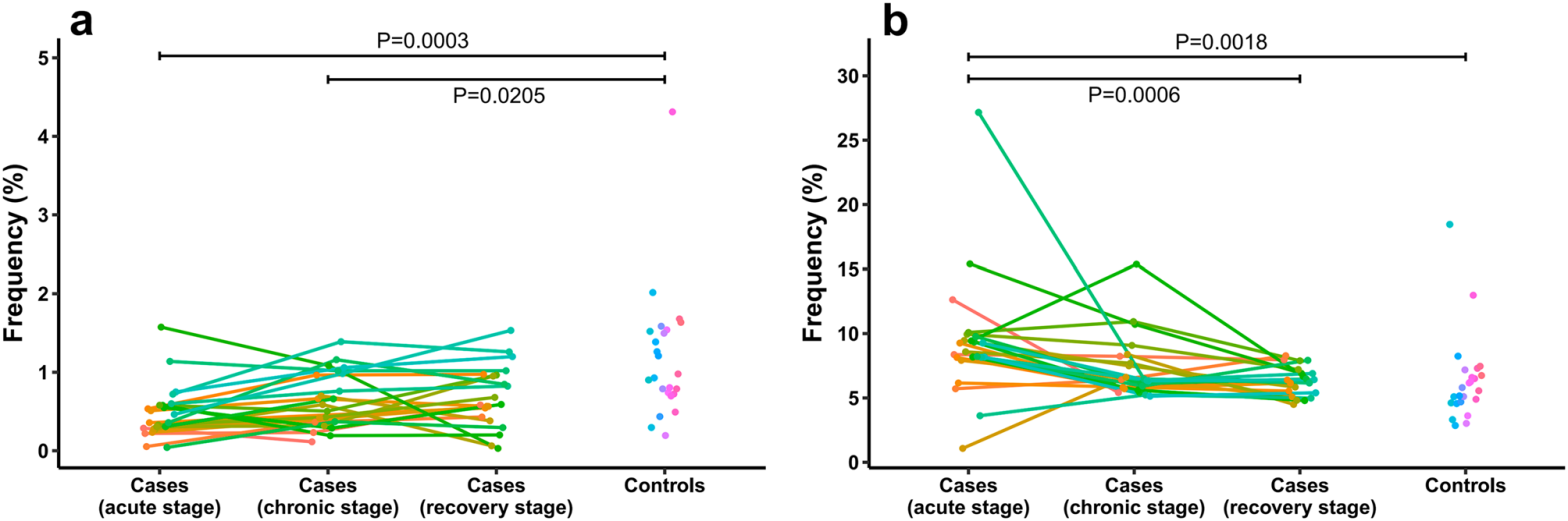
TRBV frequencies of the OMDT cases in acute, chronic, and recovery stages and of the controls. (**a**) TRBV6-4. (**b**) TRBV7-9. Dots of the same color represent TRBV frequencies of the same subject, the connecting lines indicate varying trends, and *P* values were obtained by the Man-Whitney U test with Holm’s adjustment.

After the HTS, we identified 66 TRBV subtypes, 2 TRBD subtypes, and 14 TRBJ subtypes in total. Frequency of each TRBV/TRBD/TRBJ subtype was plotted according to case in 3 stages and control, and the averages of frequency were compared between the 4 groups using the Kruskal–Wallis test with adjusted *P* = 6.1 × 10^–4^. We found that only TRBV6-4 and TRBV7-9 usage were significantly different between the 4 groups (*P* = 2.1 × 10^–4^ and *P* = 1.5 × 10^–4^, respectively). Further between-group analysis of TRBV6-4 and TRBV7-9 subtypes by the Man-Whitney U test with Holm’s adjustment revealed that TRBV6-4 frequencies of the cases in acute and chronic stages were significantly lower than the controls (*P* = 3.3 × 10^–4^ and *P* = 0.020, respectively), and TRBV7-9 frequencies of the cases in acute stage were significantly higher than the cases in recovery stage and the controls (*P* = 6.3 × 10^–4^ and *P* = 0.002, respectively) (Fig. 1). Fold changes of TRBV6-4 and TRBV7-9 frequencies in the OMDT cases relative to the controls were listed in Supplementary Table S2 online.

**Figure 2:**
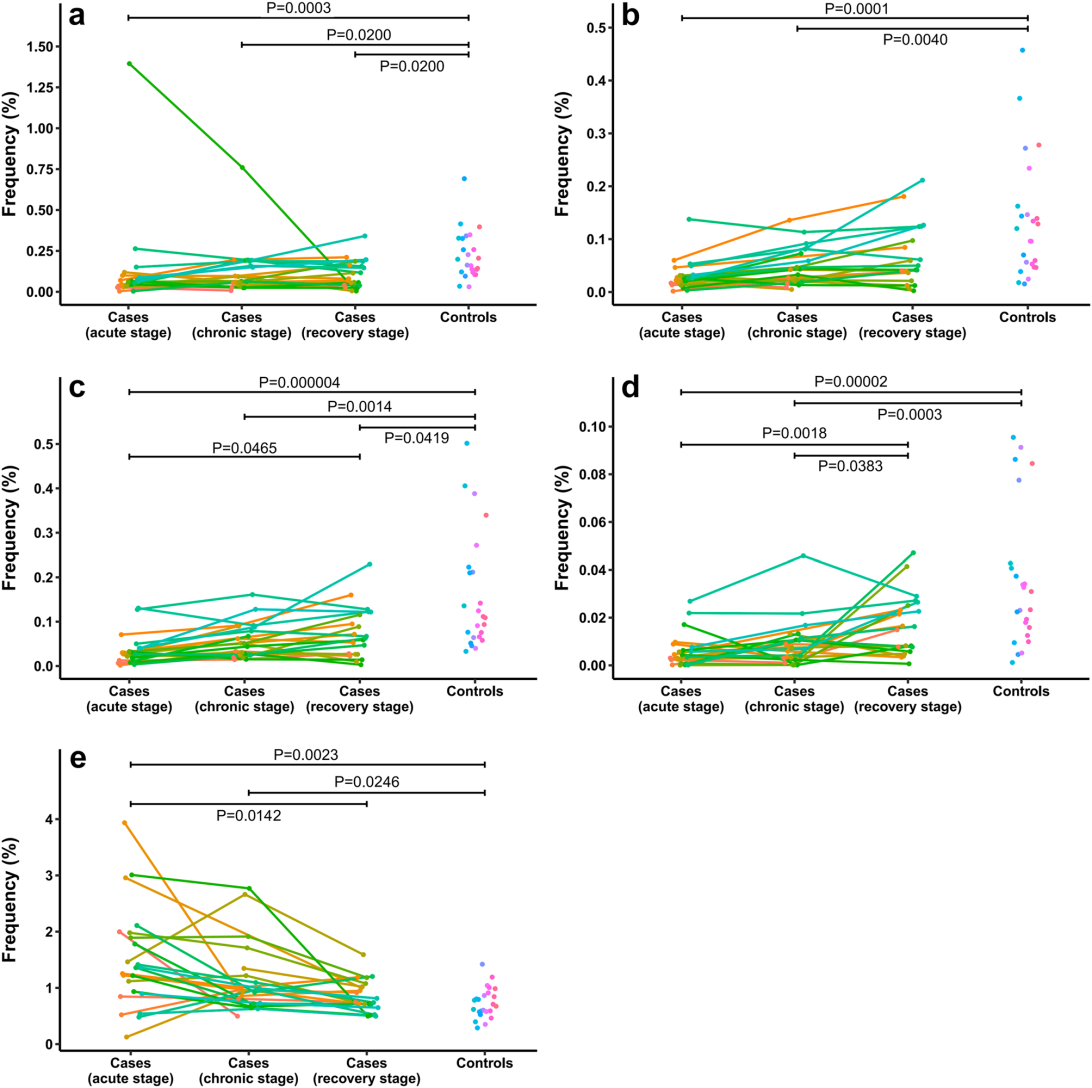
TRBV-TRBJ combination frequencies of the OMDT cases in acute, chronic, and recovery stages and of the controls. (**a**) TRBV6-4-TRBJ2-1. (**b**) TRBV6-4-TRBJ2-2. (**c**) TRBV6-4-TRBJ2-3. (**d**) TRBV6-4-TRBJ2-6. (**e**) TRBV7-9-TRBJ2-1. Dots of the same color represent TRBV-TRBJ combination frequencies of the same subject, the connecting lines indicate varying trends, and *P* values were obtained by the Man-Whitney U test with Holm’s adjustment.

TRBV-TRBJ combination is an important source of CDR3 sequence diversification. TRBV6-4/TRBV7-9 and 14 TRBJ combinations were compared between the 4 groups using the Kruskal–Wallis test with adjusted *P* = 0.001. We found that only TRBV6-4-TRBJ2-1, TRBV6-4-TRBJ2-2, TRBV6-4-TRBJ2-3, TRBV6-4-TRBJ2-6, and TRBV7-9-TRBJ2-1 combinations were significantly different between the 4 groups (*P* = 3.1 × 10^–4^, *P* = 4.1 × 10^–5^, *P* = 2.6 × 10^–6^, *P* = 5.0 × 10^–7^, and *P* = 4.2 × 10^–4^, respectively). We further conducted between-group analysis of the 5 TRBV-TRBJ combinations by the Man-Whitney U test with Holm’s adjustment. As shown in Fig. 2, TRBV6-4-TRBJ2-1 combination frequencies of the cases in acute, chronic, and recovery stages were significantly lower than the controls (*P* = 3.3 × 10^–4^, *P* = 0.020, and *P* = 0.020, respectively). TRBV6-4-TRBJ2-2 frequencies of the cases in acute and chronic stages were significantly lower than the controls (*P* = 1.1 × 10^–4^ and *P* = 0.004, respectively). TRBV6-4-TRBJ2-3 frequencies of the cases in acute, chronic, and recovery stages were significantly lower than the controls (*P* = 4.0 × 10^–6^, *P* = 0.001, and *P* = 0.042, respectively), and TRBV6-4-TRBJ2-3 frequencies of the cases in acute stage were significantly lower than the cases in recovery stage (*P* = 0.046). TRBV6-4-TRBJ2-6 frequencies of the cases in acute and chronic stages were significantly lower than the controls (*P* = 1.6 × 10^–5^ and *P* = 3.3 × 10^–4^, respectively) and the cases in recovery stage (*P* = 0.002 and *P* = 0.038, respectively). TRBV7-9-TRBJ2-1 frequencies of the cases in acute stage were significantly higher than the cases in recovery stage and the controls (*P* = 0.014 and *P* = 0.002, respectively), and TRBV7-9-TRBJ2-1 frequencies of the cases in chronic stage were also significantly higher than the controls (*P* = 0.025). Fold changes of the 5 TRBV-TRBJ combination frequencies in the OMDT cases relative to the controls were listed in Supplementary Table S2 online.

### CDR3 nucleotide and amino acid sequences

For each of the 5 significant TRBV-TRBJ combinations, there included too many CDR3-nt or CDR3-aa sequences. For example, the TRBV7-9-TRBJ2-1 combination included 205,117 CDR3-nt sequences and 174,284 CDR3-aa sequences, and most of them were of low frequency. Since the most expanded clonotypes are more likely to show association with OMDT, we only selected CDR3-nt or CDR3-aa sequences with a frequency more than 0.05% from the 5 TRBV-TRBJ combinations. Eventually, there were 8 CDR3-nt and 8 CDR3-aa sequences selected from TRBV6-4-TRBJ2-1 combination, 2 CDR3-nt and 2 CDR3-aa sequences from TRBV6-4-TRBJ2-2 combination, 6 CDR3-nt and 6 CDR3-aa sequences from TRBV6-4-TRBJ2-3 combination, 1 CDR3-nt and 1 CDR3-aa sequences from TRBV6-4-TRBJ2-6 combination, and 98 CDR3-nt and 96 CDR3-aa sequences from TRBV7-9-TRBJ2-1 combination.

After comparison of frequencies between the groups, we obtained 10 CDR3-nt and 7 CDR3-aa sequences in TRBV7-9-TRBJ2-1 combination, and 1 CDR3-nt and 1 CDR3-aa sequences in TRBV6-4-TRBJ2-1 combination, reaching the statistically significant levels. Their linear trends with decreasing OMDT severity from acute to recovery stage were also analyzed and listed in Table 3. To be noted, the 10 CDR3-nt and 7 CDR3-aa sequences in TRBV7-9-TRBJ2-1 combination were not found in the controls.

**Table 3:** OMDT severity-associated TCR β-chain CDR3 sequences.

Sequences	Percentage of frequency (%)^a^	Subjects	Estimate (SE)	*t*	*P*
**cdr3-nt**					
TRBV7-9-TRBJ2-1					
TGTGCCAGCAGGCCAACGGGGGGCCTGCGGAATGAGCAGTTCTTC	0.407	Case 3, 13, 19	− 0.266 (0.003)	− 91.8	1.2 × 10^–4^
TGTGCCAGCAGCCCACGTGGGTATGAGCAGTTCTTC	2.600	Case 5, 7, 10	− 1.732 (0.002)	− 870.8	1.3 × 10^–6^
TGTGCCAGCAGCCCCCGGGGGTATGAGCAGTTCTTC	0.149	Case 5, 7, 10	− 0.099 (0.000)	− 730.8	1.9 × 10^–6^
TGTGCCAGCAGCCCGAGAGGATATGAGCAGTTCTTC	0.117	Case 5, 7, 10	− 0.078 (0.000)	− 1350.8	5.5 × 10^–7^
TGTGCCAGCAGCCCCTTGACTAGCGGGGCGTACAATGAGCAGTTCTTC	0.053	Case 65, 66, 68	0.035 (0.000)	5429.0	1.2 × 10^–4^
TGTGCCAGCAGCTTAAGGGAAAACTCCTACAATGAGCAGTTCTTC	0.101	Case 5, 11,12	0.067 (0.000)	1094.8	8.3 × 10^–7^
TGTGCCAGCAGCGAAACTAGCGGGGGGGCCTACAATGAGCAGTTCTTC	0.121	Case 7, 9, 13, 61	0.081 (0.000)	2954.0	1.2 × 10^–7^
TGTGCCAGCAGCGGCGGACTTTCTCTGGGGAATGAGCAGTTCTTC	0.059	Case 7, 9, 13, 61	0.039 (0.000)	3603.0	7.7 × 10^–8^
TGTGCCAGCAGCTTCGTTTAGCGGGGCTGGAGCTCCTACAATGAGCAGTTCTTC	0.236	Case 7, 9, 13, 61	0.154 (0.001)	110.9	1.6 × 10^–6^
TGTGCTAGCAGCGCAGGGCAAGAGCAGTTCTTC	0.063	Case 62, 63 64, 68	0.042 (0.000)	434.1	5.3 × 10^–6^
TRBV6-4-TRBJ2-1					
TGTGCCAGCTCTTCTTCCGTTAGCTCCTACAATGAGCAGTTCTTC	0.865	Case 66,93,95,96 Control 104,117,118,120,121,123,124	− 0.045 (0.007)	− 6.8	8.0 × 10^–5^
**cdr3-aa**					
TRBV7-9-TRBJ2-1					
CASRPTGGLRNEQFF	0.407	Case 3, 13, 19	− 0.266 (0.003)	− 91.8	1.2 × 10^–4^
CASSPLTSGAYNEQFF	0.053	Case 65, 66, 68	0.035 (0.000)	5429.0	1.2 × 10^–4^
CASSLRENSYNEQFF	0.101	Case 5,11,12,13,19	0.067 (0.000)	647.9	3.4 × 10^–11^
CASSETSGGAYNEQFF	0.121	Case 7, 9, 13, 61	0.081 (0.000)	2954.0	1.2 × 10^–7^
CASSGGLSLGNEQFF	0.059	Case 7, 9, 13, 61	0.039 (0.000)	3603.0	7.7 × 10^–8^
CASSFV*RGWSSYNEQFF	0.236	Case 7, 9, 13, 61	0.154 (0.002)	102.6	2.0 × 10^–6^
CASSAGQEQFF	0.067	Case 62, 63 64, 68	0.044 (0.000)	441.2	5.1 × 10^–6^
TRBV6-4-TRBJ2-1					
CASSSSVSSYNEQFF	0.865	Case 66,93,95,96 Control 104,117,118,120,121,123,124	− 0.045 (0.007)	− 6.8	8.0 × 10^–5^

OMDT, occupational medicamentosa-like dermatitis due to trichloroethylene; SE, standard error.

Linear regression of sequence frequencies with decreasing OMDT severity from acute to recovery stage.

^a^Percentage of frequency of the sequence in the total frequency of corresponding TRBV-TRBJ combination.

## Discussion

OMDT has been regarded as a T cell-mediated, Type IV hypersensitivity reaction^2,4^, so T cells may play a pivotal role in the development of OMDT. TCR is the key to initiate T cell reactions, therefore, studying TCR is of great significance for the treatment and prevention of OMDT. Previously, we had explored cross-sectionally the characteristics of TCR repertoires from OMDT patients^8^; the dynamic features of OMDT TCR repertoires were further analyzed in the present study.

It is known that OMDT is a kind of hypersensitivity disease, the occurrence of OMDT is not related to the exposure dose of trichloroethylene^2^. Although the average trichloroethylene exposure concentration was higher in the cases, there were cases exposed to trichloroethylene concentration much lower than the controls. All the controls had been exposed to trichloroethylene for more than half a year, and their average trichloroethylene exposure period was significantly longer than the cases.

The staging of OMDT was based on the main clinical manifestation and auxiliary laboratory examination of the cases^9^. It is noticeable that most of the laboratory indices in the cases in acute stage significantly deviated from those in the controls, and generally returned in recovery stage to the reference levels as in the controls (Table 2), suggesting a decreasing severity of OMDT from acute to recovery stage.

Dynamic analysis of OMDT TCR repertoires started with the repertoire diversity. As reported previously in the pilot cross-sectional study^8^, we found no significant difference in CDR3 repertoire diversity measured by 6 indices between the cases and the controls, although decreased diversity has been observed in similar diseases^10–12^. Remarkably, the 6 indices all showed an increasing trend with the decreasing severity from acute to recovery stage in the cases, however, the differences between the stages were not statistically significant.

We next examined TRBV/TRBD/TRBJ usage and combination, and found that only TRBV6-4 and TRBV7-9 usage were significantly associated with OMDT, and 5 of their combinations with TRBJ were further identified to be OMDT severity-related. In the previous cross-sectional study^8^, we also identified that TRBV6-4 frequencies of the cases were significantly lower than the controls (*P* < 0.05), and TRBV7-9 frequencies were higher in the cases, but the difference with the controls was not statistically significant. It seems that the time point of repertoire detection is quite critical, because we found in this longitudinal study that only the cases in acute stage showed significantly higher TRBV7-9 frequencies than the controls, suggesting the importance of dynamic analysis in studies of TCR repertoire. Moreover, decreased frequencies of TRBV6-4-TRBJ2-1, TRBV6-4-TRBJ2-2, TRBV6-4-TRBJ2-3, and TRBV6-4-TRBJ2-6 combinations in the cases in the previous cross-sectional study were also validated in the present study, and we further observed their restoring trends from acute to recovery stage by the dynamic analysis. Other significant TRBV usage such as TRBV7-6 and TRBV4-1, and TRBV-TRBJ combinations such as TRBV7-6-TRBJ2-5 and TRBV4-1-TRBJ2-5 from the previous study were not re-identified, because some of the prior positive results were possibly obtained by chance due to the cross-sectional design, and some of them still need to be further validated with larger sample size.

In the next step, we further analyzed part of CDR3-nt and CDR3-aa sequences with relatively high frequency from the 5 significant TRBV-TRBJ combinations. We finally identified 10 CDR3-nt sequences and 7 CDR3-aa sequences in TRBV7-9-TRBJ2-1 combination and 1 CDR3-nt sequence and 1 CDR3-aa sequence in TRBV6-4-TRBJ2-1 combination, of which the frequencies significantly varied with the severity of OMDT. Because these sequences were not commonly shared between the cases, none of them was previously found in the cross-sectional study^8^.

We observed opposite trends in the analysis of TRBV/TRBD/TRBJ usage and combination and CDR3 sequence frequency with varying severity of OMDT from acute to recovery stage. The decreasing trends, such as in the CDR3-nt sequence ‘TGTGCCAGCAGGCCAACGGGGGGCCTGCGGAATGAGCAGTTCTTC’ and CDR3-aa sequence ‘CASRPTGGLRNEQFF’, indicated that the corresponding T cells might be effector cells. They could rapidly expand when OMDT occurred, and gradually fell back to normal levels as the disease was cured. On the other hand, the increasing trends, such as in the CDR3-nt sequence ‘TGTGCCAGCAGCCCCTTGACTAGCGGGGCGTACAATGAGCAGTTCTTC’ and CDR3-aa sequence ‘CASSPLTSGAYNEQFF’, indicated that the corresponding T cells might be regulatory cells (Tregs), which are as important as effector cells in the pathology of hypersensitivity disease^13,14^. The assumption was based on the animal experiment report that TCE caused imbalance of T helper cell subsets in BALB/c mice^15^, and more importantly, on the clinical evidence that Treg ratios significantly decreased in OMDT in the acute stage^16^. Those Tregs could be first inhibited when OMDT occurred, and gradually restored to normal levels with the recovery from the disease.

Additionally, we analyzed the potential public T cell clonotypes associated with OMDT by comparing Jaccard similarity indices between the cases in different stages and the controls. As shown in Supplementary Fig. S2 online, Jaccard similarity indices calculated based on CDR3-aa sequences in the cases in acute stage and in recovery stage were significantly higher than in the controls (both *P* < 0.01), but Jaccard similarity indices calculated based on CDR3-nt sequences were not significantly different between the cases and controls. The results suggested that the situation of public T cell clonotypes was similar to the previous study^8^, and that the possibility of public T cell clonotypes associated with OMDT was relatively small.

Susceptibility of OMDT had been linked to HLA-B*13:01 genotype in the previous literature^17,18^, so we tried to screen the TCR β CDR3 repertoire under the same HLA-B*13:01 background in the previous case–control study^8^. Unexpectedly, different TCR clonotypes were found specific to OMDT cases carrying the same HLA-B*13:01 allele, and there were also patients carrying other alleles such as HLA-B*08:01, HLA-B*44:03, HLA-B*46:01, and HLA-B*07:02, etc., which suggest that different mechanisms of immune recognition might occur in OMDT. Considering the complexity of OMDT mechanism, we focused only on the dynamic change of TCR repertoires with the disease severity in the present study, leaving the mechanisms of immune recognition in OMDT to be further discussed in the future studies.

Taken together, the present study, being an important sequel to the previous cross-sectional study, further explored in depth the dynamic characteristics of OMDT TCR repertoires. Not only did it validate the association of some previously found TRBV and TRBV-TRBJ combinations with OMDT, but also it discovered how TCR repertoires vary with the severity in the course of OMDT. Moreover, identification of specific, severity-related TCRs may provide important therapeutic targets as well as monitoring indicators for OMDT in clinical practice. Because of the dynamic features of TCR repertoires, the time point of repertoire detection is vitally important. Limited by the samples, we only detected TCR repertoires at one time point in each of the stages for each case, which hindered us from outlining the panorama of the whole OMDT TCR repertoire. Decreased repertoire diversity, highly expanded T cell clonotypes, more severity-associated TCRs, and other new features of TCR repertoires might be further uncovered by multiple time-point longitudinal studies in the future.

## Methods

### Cases and controls

Twenty-four OMDT patients, who were successively admitted to Shenzhen Prevention and Treatment Center for Occupational Diseases (SPTCOD) from January 2014 to December 2018, were recruited as cases. A panel of at least three occupational physicians diagnosed their diseases based on the diagnostic criteria GBZ 185–2006 issued by Ministry of Health of the People’s Republic of China^19^. The clinical courses of the cases all ended before June 2019. Twenty-four healthy workers, who had been exposed to trichloroethylene during their work for more than half a year, were randomly recruited as controls when they received health check in SPTCOD from January 2017 to June 2019.

We collected detailed information on demographic characteristics, occupational history and medical conditions from the 24 OMDT cases and 24 controls. We also recorded clinical data of the cases during their hospitalization including main clinical manifestation and auxiliary examination results. The clinical courses of the cases were divided into 3 stages, namely acute stage, chronic stage (or early period of chronic stage), and recovery stage (or later period of chronic stage), according to the clustering of the clinical manifestation and laboratory examination indices during the hospitalization, which has be previously reported^9^.

Written informed consent for study participation was obtained from all subjects or their guardians. The use of peripheral blood samples for further studies beyond routine laboratory examination was approved by the Ethics Committee of SPTCOD (No. LL-201802). This study abides by the Helsinki Declaration on ethical principles for medical research involving human subjects.

### Blood samples collection and preparation

Peripheral blood samples of the cases were collected after their routine laboratory examination during the hospitalization, and stored at -80℃ for later use. One sample in each of the 3 stages of each case was selected for DNA extraction and the deep sequencing. A few samples were missing in the stages, thus there were 23, 22 and 21 samples selected in the acute, chronic and recovery stages, respectively. The average time period for samples collection was 26.35 ± 43.94 days between the acute and chronic stages, and 81.78 ± 67.77 days between the chronic and recovery stages. Blood samples of the 24 controls were all collected during their health check.

Genomic DNA was extracted from about 2.0 mL of each blood sample using a QIAamp DNA Blood Midi Kit (QIAGEN, Shanghai, China) following the manufacturer’s instructions. DNA concentration was quantified using a Qubit 2.0 Fluorometer (Invitrogen, Carlsbad, CA) and DNA integrity was tested by the agarose gel electrophoresis.

### Multiplex-PCR amplification of the TCR β CDR3 region

According to the IMGT unique numbering for V-DOMAIN^20^, the TCR β CDR3 region starts with the second conserved cysteine (position 104) encoded by the Vb gene segment and ends with the conserved phenylalanine or tryptophan (position 118) encoded by the Jb gene segment. A multiplex-PCR system was designed to amplify rearranged TCR β CDR3 regions from genomic DNA using a set of forward and reverse primers to generate the template library for Genome Analyzer. The primers and PCR conditions had been reported before^21,22^. After the amplification and agarose gel electrophoresis selection, the PCR products were purified and quantitated by determining the average molecule length using the Agilent 2100 bioanalyzer instrument (Agilent DNA 1000 Reagents) and by real-time quantitative PCR (QPCR) (TaqMan Probe).

### High-throughput sequencing and data analysis

As previously reported^8^, the libraries obtained from the last step were amplified with cBot to generate the cluster on the flow cell, and the amplified flow cell was pair-end sequenced using a Hiseq4000 instrument, with a read length of 100 as the most frequently used sequencing strategy. The acquisition of raw data of sequences, basic data processing and sequence alignment were as previously described by Bolotin et al.^23^. Thereafter, the V, D, J gene segments and CDR3 regions were identified, and nucleotides were translated into amino acids^8^.

The sequencing data were sorted and calculated into the following indices: (1) repertoire diversity indices including resampled diversity, extrapolate diversity, chaoE, chao1, Shannon–Wiener index, and inverse Simpson index, (2) TRBV/TRBD/TRBJ usage and combination, (3) CDR3-nt and CDR3-aa sequence frequency. Resampled diversity and extrapolate diversity are clonal diversity under the same amount of effective data. Resampled diversity uses the lowest amount of data, while extrapolate diversity uses the maximum amount of data^24^. ChaoE is the clone diversity obtained after normalizing the amount of clone data, and chao1, also known as chao estimate, is the predicted clone diversity under the assumption that the amount of data is completely saturated^24^. Shannon–Wiener index and inverse Simpson index are both commonly used measures of diversity, which have been introduced previously^8^.

### Statistical analysis

The statistical analyses and graphing were conducted with R v. 3.6.3^25^. Numerical variables were presented as mean with standard deviation (*SD*) if they were normally distributed, otherwise as median (*M*) with median absolute deviation (*MAD*). Repertoire diversity indices, frequencies of TRBV, TRBD, TRBJ, and TRBV-TRBJ combination, and frequencies of CDR3-nt and CDR3-aa sequences were compared between different stages of the cases or between the cases in different stages and the controls using the Man-Whitney U test with Holm’s adjustment. The trend of CDR3-nt or CDR3-aa sequence frequencies with decreasing OMDT severity from acute to recovery stage was analyzed by the linear regression model. Categorical variables such as the percentage of male and of Han were compared between the cases and controls using *χ*^2^-test or Fisher’s exact test accordingly. All tests were two-sided and *P* < 0.05 was considered statistically significant unless it was adjusted in multiple comparisons.

### Ethics approval

All procedures performed in the study involving human participants were in accordance with the ethical standards of the Ethics Committee of Shenzhen Prevention and Treatment Center for Occupational Diseases and with the 1975 Declaration of Helsinki and its later amendments or comparable ethical standards. The study was approved by the Ethics Committee of Shenzhen Prevention and Treatment Center for Occupational Diseases (No. LL-201802).

### Consent to participate

Informed consent was obtained from all individual participants included in the study or their legal guardians.

## Supplementary Information

Supplementary Information 1.

Supplementary Information 2.

Supplementary Information 3.

Supplementary Information 4.

## Data Availability

The data that support the findings of this study are available from the corresponding authors upon reasonable request.
