# Supplementary material for: Dynamic analysis of peripheral blood TCR β-chain CDR3 repertoire in occupational medicamentosa-like dermatitis due to trichloroethylene
Source: Sci Rep. 2021 May 11;11:9971. doi: 10.1038/s41598-021-89431-w (PMC8113444; doi:10.1038/s41598-021-89431-w)
Supplement: Supplementary file 1 — Supplementary Information 1. [file 41598_2021_89431_MOESM1_ESM.pdf]

Article title: Dynamic analysis of peripheral blood TCR β-chain CDR3 repertoire in occupational medicamentosa-like dermatitis due to trichloroethylene

Journal name: Scientific Reports

Author names: Dafeng Lin[1], Dianpeng Wang[1], Peimao Li[1], Xiangli Yang[1], Wei Liu[2], Lu Huang[3], Zhimin Zhang[1], Yanfang Zhang[1], Wen Zhang[1], Naixing Zhang[1], Ming Zhang[1], and Xianqing Huang[1]

Affiliation: [1] Medical Laboratory, Shenzhen Prevention and Treatment Center for Occupational Diseases, Shenzhen 518020, China; [2] Key Laboratory of Modern Toxicology of Shenzhen, Medical Key Laboratory of Guangdong Province, Medical Key Laboratory of Health Toxicology of Shenzhen, Shenzhen Center for Disease Control and Prevention, Shenzhen 518055, China; [3]Fuyong Prevention and Health Care Center, Bao'an District, Shenzhen 518103, China

E-mail address of the corresponding author: david1385@foxmail.com

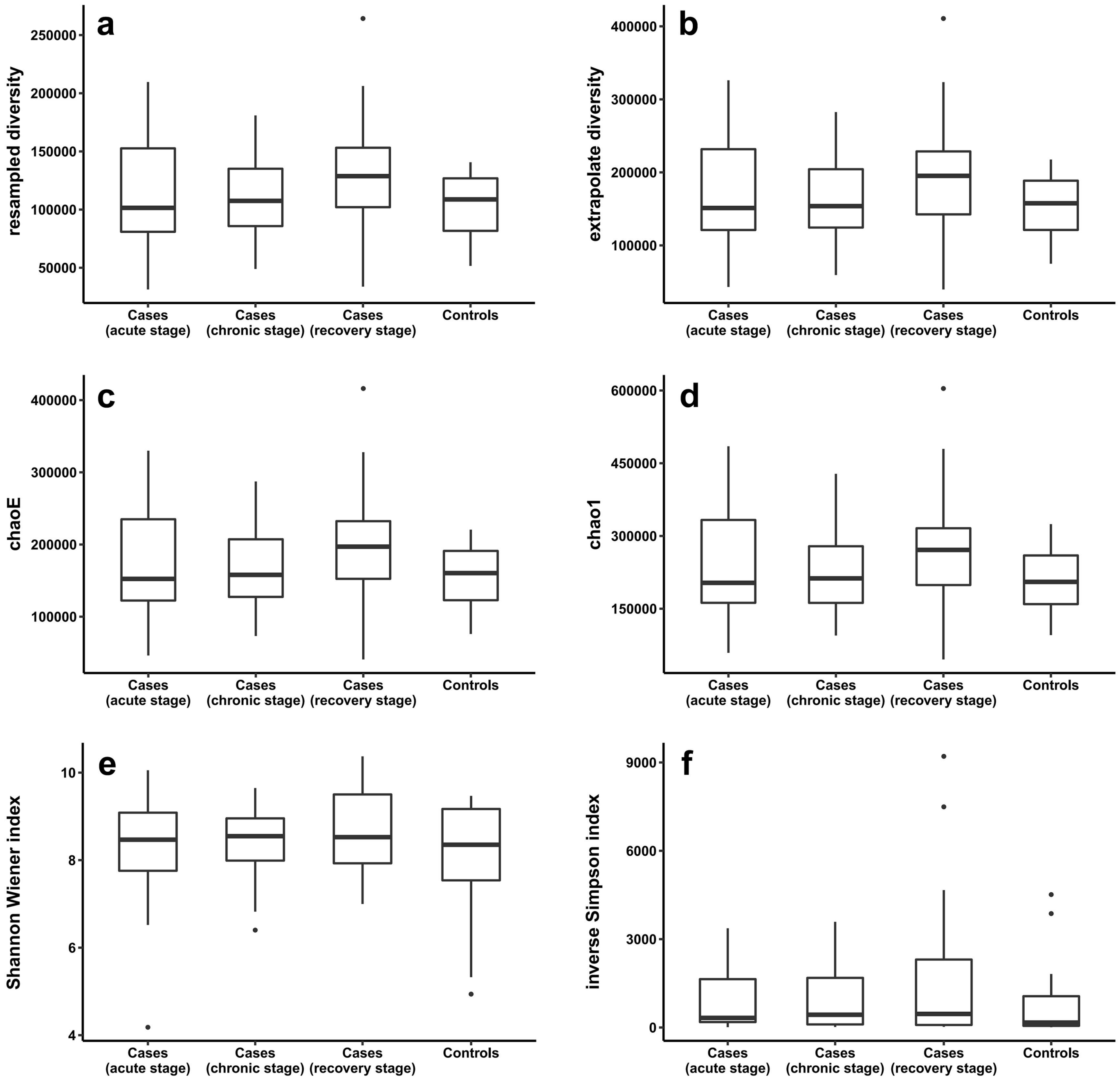

Supplementary Figure S1. TCR repertoire diversity of the OMDT cases in acute, chronic, and recovery stages and of the controls. **a** Resampled diversity. **b** Extrapolate diversity. **c** ChaoE. **d** Chao1. **e** Shannon-Wiener index. **f** Inverse Simpson index. The middle lines inside the boxes represent medians, the bottom and top of the boxes represent first and third quartiles, respectively, the whiskers extend to the furthest data point within 1.5 times the inter quartile range, and the dots past the ends of the whiskers are outliers.
