# Supplementary material for: Dynamic analysis of peripheral blood TCR β-chain CDR3 repertoire in occupational medicamentosa-like dermatitis due to trichloroethylene
Source: Sci Rep. 2021 May 11;11:9971. doi: 10.1038/s41598-021-89431-w (PMC8113444; doi:10.1038/s41598-021-89431-w)
Supplement: Supplementary file 2 — Supplementary Information 2. [file 41598_2021_89431_MOESM2_ESM.pdf]

Article title: Dynamic analysis of peripheral blood TCR β-chain CDR3 repertoire in occupational medicamentosa-like dermatitis due to trichloroethylene

Journal name: Scientific Reports

Author names: Dafeng Lin[1], Dianpeng Wang[1], Peimao Li[1], Xiangli Yang[1], Wei Liu[2], Lu Huang[3], Zhimin Zhang[1], Yanfang Zhang[1], Wen Zhang[1], Naixing Zhang[1], Ming Zhang[1], and Xianqing Huang[1]

Affiliation: [1] Medical Laboratory, Shenzhen Prevention and Treatment Center for Occupational Diseases, Shenzhen 518020, China; [2] Key Laboratory of Modern Toxicology of Shenzhen, Medical Key Laboratory of Guangdong Province, Medical Key Laboratory of Health Toxicology of Shenzhen, Shenzhen Center for Disease Control and Prevention, Shenzhen 518055, China; [3]Fuyong Prevention and Health Care Center, Bao'an District, Shenzhen 518103, China

E-mail address of the corresponding author: david1385@foxmail.com

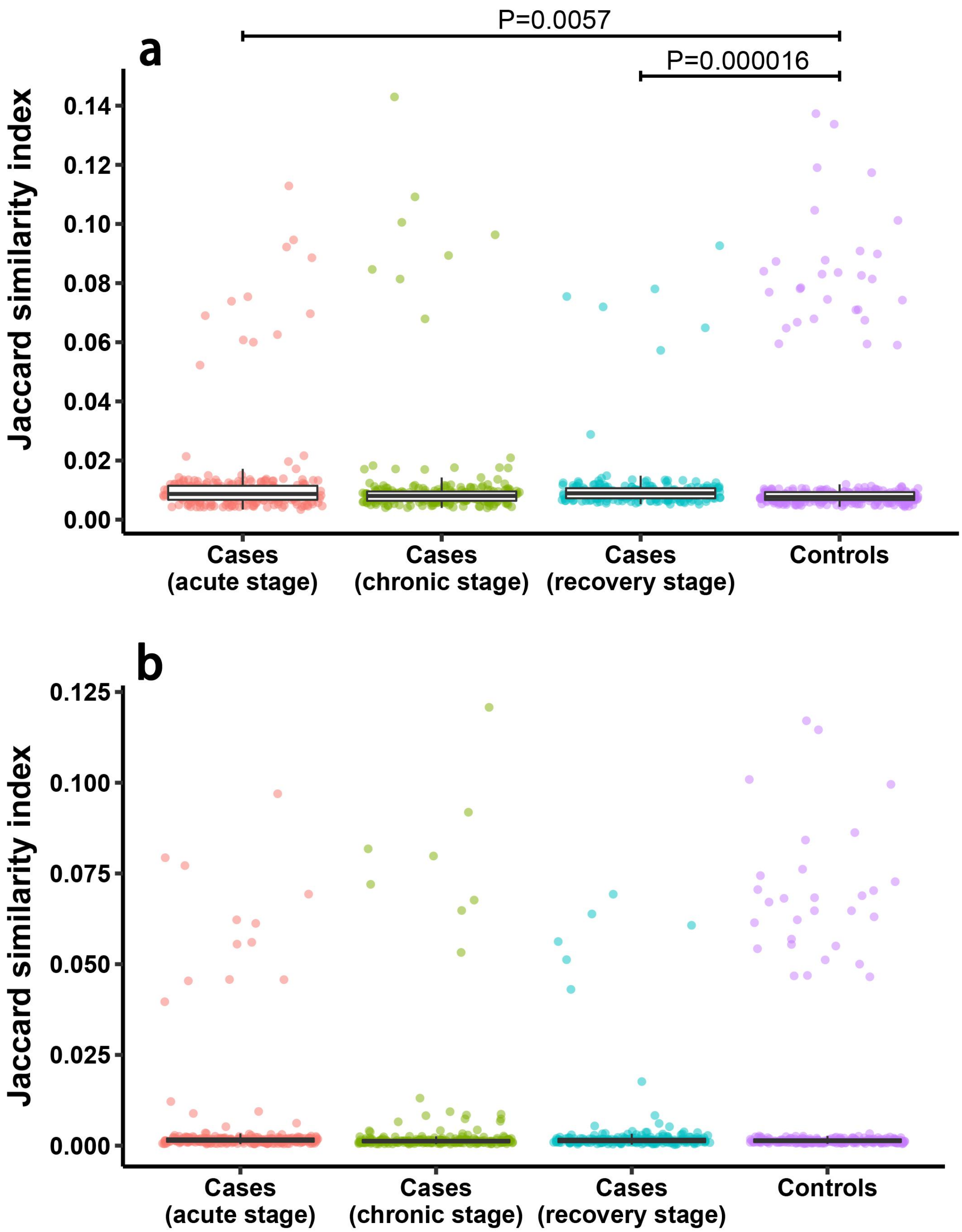

Supplementary Figure S2. Jaccard similarity indices of the OMDT cases in acute, chronic, and recovery stages and of the controls. **a** CDR3-aa sequences. **b** CDR3-nt sequences. Each dot represents the Jaccard similarity index comparing all CDR3 sequences from two subjects in the same group. The middle lines inside the boxes represent medians, the bottom and top of the boxes represent first and third quartiles, respectively, and the whiskers extend to the furthest data point within 1.5 times the inter quartile range. *P* values were obtained by the Man-Whitney U test with Holm's adjustment.
