# Supplementary material for: Dynamic analysis of peripheral blood TCR β-chain CDR3 repertoire in occupational medicamentosa-like dermatitis due to trichloroethylene
Source: Sci Rep. 2021 May 11;11:9971. doi: 10.1038/s41598-021-89431-w (PMC8113444; doi:10.1038/s41598-021-89431-w)
Supplement: Supplementary file 3 — Supplementary Information 3. [file 41598_2021_89431_MOESM3_ESM.pdf]

**Article title:** Dynamic analysis of peripheral blood TCR β-chain CDR3 repertoire in occupational medicamentosa-like dermatitis due to trichloroethylene

**Journal name:** Scientific Reports

**Author names:** Dafeng Lin[1], Dianpeng Wang[1], Peimao Li[1], Xiangli Yang[1], Wei Liu[2], Lu Huang[3], Zhimin Zhang[1], Yanfang Zhang[1], Wen Zhang[1], Naixing Zhang[1], Ming Zhang[1], and Xianqing Huang[1]

**Affiliation:** [1] Medical Laboratory, Shenzhen Prevention and Treatment Center for Occupational Diseases, Shenzhen 518020, China; [2] Key Laboratory of Modern Toxicology of Shenzhen, Medical Key Laboratory of Guangdong Province, Medical Key Laboratory of Health Toxicology of Shenzhen, Shenzhen Center for Disease Control and Prevention, Shenzhen 518055, China; [3]Fuyong Prevention and Health Care Center, Bao'an District, Shenzhen 518103, China

**E-mail address of the corresponding author:** david1385@foxmail.com

**Supplementary Table S1.** The overview of TCR β-chain CDR3 sequence statistics.

| sampleID | sequencingID | group | Raw reads | Raw bases (Mb) | Clean reads | Clean bases (Mb) | Clean data rate (%) | Clean read Q20 (%) | Clean read Q30 (%) | GC content (%) | TCR reads | Clonetypes |
|---|---|---|---|---|---|---|---|---|---|---|---|---|
| B9393 | B9393-119-0 | Control | 16328318 | 2449.25 | 16135056 | 2196.39 | 89.68 | 98.41 | 95.56 | 56.09 | 7427305 | 159803 |
| B10968 | B10968-105-0 | Control | 16327884 | 2449.18 | 16072876 | 2280.43 | 93.11 | 97.47 | 93.19 | 56.59 | 7128850 | 90616 |
| B9315 | B9315-104-0 | Control | 16328188 | 2449.23 | 16091202 | 2217.43 | 90.54 | 98.49 | 95.72 | 56.15 | 7395382 | 113229 |
| B4293 | B4293-101-0 | Control | 16328184 | 2449.23 | 16151388 | 2248.24 | 91.79 | 97.36 | 92.91 | 55.87 | 7539183 | 76244 |
| B8953 | B8953-102-0 | Control | 16328258 | 2449.24 | 16146802 | 2127.24 | 86.85 | 98.29 | 95.27 | 56.88 | 5785326 | 127816 |
| B10970 | B10970-106-0 | Control | 16328168 | 2449.23 | 16125440 | 2176.10 | 88.85 | 98.29 | 95.33 | 55.48 | 7628061 | 124391 |
| B9375 | B9375-120-0 | Control | 16092578 | 2413.89 | 15838364 | 2131.91 | 88.32 | 97.84 | 94.93 | 55.93 | 7355130 | 214173 |
| B11117 | B11117-110-0 | Control | 16328486 | 2449.27 | 16078574 | 2209.65 | 90.22 | 99.48 | 98.43 | 56.18 | 7557003 | 158890 |
| B9373 | B9373-121-0 | Control | 16327774 | 2449.17 | 16111742 | 2242.44 | 91.56 | 98.78 | 96.64 | 56.52 | 7570197 | 205827 |
| B11201 | B11201-115-0 | Control | 16328682 | 2449.30 | 16045882 | 2203.82 | 89.98 | 99.47 | 98.39 | 55.93 | 7574190 | 185536 |
| B10987 | B10987-109-0 | Control | 16328182 | 2449.23 | 16020740 | 2230.52 | 91.07 | 98.10 | 94.86 | 56.33 | 7376885 | 119062 |
| B10512 | B10512-111-0 | Control | 16328234 | 2449.24 | 16012050 | 2208.09 | 90.15 | 97.56 | 93.50 | 56.17 | 7331108 | 167666 |
| B10971 | B10971-107-0 | Control | 16328280 | 2449.24 | 16085532 | 2211.39 | 90.29 | 98.30 | 95.35 | 54.81 | 7628894 | 140382 |

| | | | | | | | | | | | | |
|---|---|---|---|---|---|---|---|---|---|---|---|---|
| B9419 | B9419-118-0 | Control | 16328208 | 2449.23 | 16147064 | 2264.80 | 92.47 | 98.06 | 94.66 | 55.99 | 7501469 | 145909 |
| B4022 | B4022-122-0 | Control | 16327026 | 2449.05 | 15788920 | 2148.16 | 87.71 | 99.21 | 97.62 | 56.25 | 7286821 | 201873 |
| B8965 | B8965-123-0 | Control | 16328020 | 2449.20 | 16014276 | 2213.68 | 90.38 | 98.80 | 96.69 | 56.14 | 7466770 | 198011 |
| B10986 | B10986-108-0 | Control | 16328090 | 2449.21 | 16130518 | 2164.39 | 88.37 | 98.32 | 95.40 | 56.00 | 7610889 | 146758 |
| B9417 | B9417-117-0 | Control | 16328296 | 2449.24 | 16146036 | 2211.95 | 90.31 | 98.42 | 95.58 | 56.23 | 7617282 | 188326 |
| B11047 | B11047-114-0 | Control | 16328408 | 2449.26 | 15981420 | 2204.28 | 90.00 | 99.44 | 98.34 | 55.70 | 7470277 | 183127 |
| B9378 | B9378-124-0 | Control | 16327700 | 2449.16 | 16085450 | 2198.83 | 89.78 | 98.81 | 96.75 | 56.24 | 7597543 | 218850 |
| B9029 | B9029-103-0 | Control | 16328322 | 2449.25 | 16177620 | 2287.65 | 93.40 | 98.09 | 94.80 | 55.88 | 7655989 | 109517 |
| B11046 | B11046-113-0 | Control | 16328260 | 2449.24 | 16096414 | 2099.06 | 85.70 | 98.34 | 95.42 | 57.11 | 7577430 | 80597 |
| B10739 | B10739-116-0 | Control | 16328130 | 2449.22 | 16101832 | 2212.85 | 90.35 | 97.55 | 93.41 | 56.53 | 7575273 | 206539 |
| B10513 | B10513-112-0 | Control | 16328136 | 2449.22 | 15943760 | 2208.52 | 90.17 | 97.54 | 93.44 | 55.97 | 7416485 | 170432 |
| T1082 | T1082-3-1 | Case in acute stage | 16328872 | 2449.33 | 16073912 | 2192.26 | 89.50 | 99.06 | 97.33 | 56.39 | 7494941 | 211961 |
| T1422 | T1422-5-1 | Case in acute stage | 16328648 | 2449.30 | 16156006 | 2169.53 | 88.58 | 99.02 | 97.19 | 55.35 | 7700597 | 247188 |
| T1243 | T1243-7-1 | Case in acute stage | 16328898 | 2449.33 | 16028144 | 2085.28 | 85.14 | 98.84 | 96.88 | 56.53 | 7660413 | 152565 |
| T1022 | T1022-8-1 | Case in acute stage | 16328936 | 2449.34 | 16017458 | 2212.14 | 90.32 | 99.03 | 97.25 | 56.38 | 7458290 | 131566 |
| B3838 | B3838-64-1 | Case in acute stage | 16327908 | 2449.19 | 16172022 | 2206.71 | 90.10 | 98.89 | 96.91 | 56.97 | 7613911 | 303298 |

| | | | | | | | | | | | | |
|---|---|---|---|---|---|---|---|---|---|---|---|---|
| T1157 | T1157-19-1 | Case in acute stage | 16328852 | 2449.33 | 16081946 | 2224.21 | 90.81 | 98.97 | 97.03 | 56.19 | 7551497 | 149314 |
| B4529 | B4529-67-1 | Case in acute stage | 16328164 | 2449.22 | 16170376 | 2294.93 | 93.70 | 96.99 | 92.03 | 56.48 | 7357894 | 82635 |
| T1400 | T1400-9-1 | Case in acute stage | 16329004 | 2449.35 | 16222962 | 2220.31 | 90.65 | 98.59 | 96.30 | 55.58 | 7647584 | 259482 |
| B3986 | B3986-65-1 | Case in acute stage | 16328160 | 2449.22 | 16197848 | 2217.33 | 90.53 | 96.80 | 91.62 | 56.31 | 7526510 | 206899 |
| B3145 | B3145-62-1 | Case in acute stage | 16327596 | 2449.14 | 15992836 | 2208.44 | 90.17 | 98.80 | 96.69 | 56.06 | 7463837 | 121688 |
| T1008 | T1008-10-1 | Case in acute stage | 16328534 | 2449.28 | 16102806 | 2214.42 | 90.41 | 98.94 | 96.93 | 56.91 | 7610078 | 219335 |
| T1301 | T1301-11-1 | Case in acute stage | 16328658 | 2449.30 | 16059538 | 2146.86 | 87.65 | 99.02 | 97.18 | 56.44 | 7619524 | 253991 |
| B9409 | B9409-95-1 | Case in acute stage | 16327658 | 2449.15 | 16160384 | 2205.70 | 90.06 | 99.21 | 97.61 | 56.00 | 7617325 | 327933 |
| B4535 | B4535-66-1 | Case in acute stage | 16328108 | 2449.22 | 16171698 | 2236.45 | 91.31 | 98.04 | 94.64 | 55.86 | 7366418 | 141408 |
| B5463 | B5463-68-1 | Case in acute stage | 16328068 | 2449.21 | 16227182 | 2136.79 | 87.24 | 97.85 | 94.13 | 56.42 | 7782507 | 123562 |
| T1295 | T1295-12-1 | Case in acute stage | 16328656 | 2449.30 | 16117392 | 2179.69 | 88.99 | 99.03 | 97.22 | 55.03 | 7671034 | 59880 |
| B8917 | B8917-93-1 | Case in acute stage | 16328032 | 2449.20 | 16147348 | 2300.70 | 93.94 | 98.77 | 96.59 | 57.01 | 7524460 | 137882 |
| B7639 | B7639-70-1 | Case in acute stage | 16328112 | 2449.22 | 16101434 | 2254.21 | 92.04 | 97.84 | 94.12 | 57.08 | 6682464 | 43913 |
| B9408 | B9408-96-1 | Case in acute stage | 16327584 | 2449.14 | 16150864 | 2153.76 | 87.94 | 99.31 | 97.88 | 56.13 | 7635021 | 233194 |
| B8939 | B8939-94-1 | Case in acute stage | 16328226 | 2449.23 | 16173222 | 2212.60 | 90.34 | 98.32 | 95.32 | 56.41 | 7721981 | 57295 |
| B3644 | B3644-63-1 | Case in acute stage | 16328342 | 2449.25 | 16190644 | 2212.77 | 90.34 | 99.28 | 97.82 | 56.25 | 7512784 | 232219 |

| | | | | | | | | | | | | |
|---|---|---|---|---|---|---|---|---|---|---|---|---|
| B2304 | B2304-61-1 | Case in acute stage | 16328846 | 2449.33 | 16191858 | 2178.54 | 88.94 | 98.72 | 96.58 | 56.48 | 7560101 | 233037 |
| B7485 | B7485-69-1 | Case in acute stage | 16328274 | 2449.24 | 16198436 | 2226.33 | 90.90 | 97.97 | 94.45 | 56.20 | 7672211 | 116110 |
| T1140 | T1140-3-2 | Case in chronic stage | 16328666 | 2449.30 | 16005786 | 2078.86 | 84.88 | 99.03 | 97.20 | 55.75 | 7667660 | 174577 |
| T1424 | T1424-5-2 | Case in chronic stage | 16328476 | 2449.27 | 16040808 | 2144.81 | 87.57 | 98.97 | 97.04 | 55.41 | 7558569 | 284411 |
| T1248 | T1248-7-2 | Case in chronic stage | 16328890 | 2449.33 | 16147594 | 2204.55 | 90.01 | 98.69 | 96.50 | 56.57 | 7619764 | 222580 |
| T1050 | T1050-8-2 | Case in chronic stage | 16328936 | 2449.34 | 15865276 | 2196.03 | 89.66 | 98.95 | 97.05 | 56.30 | 7195248 | 152136 |
| B3943 | B3943-64-2 | Case in chronic stage | 16328574 | 2449.29 | 16069400 | 2203.64 | 89.97 | 98.91 | 96.87 | 56.31 | 7598076 | 186490 |
| T1164 | T1164-19-2 | Case in chronic stage | 16328856 | 2449.33 | 16030900 | 2183.83 | 89.16 | 98.97 | 97.02 | 55.93 | 7482998 | 158518 |
| B10715 | B10715-67-2 | Case in chronic stage | 16328174 | 2449.23 | 16086912 | 2233.35 | 91.19 | 97.69 | 93.78 | 56.20 | 7579743 | 152920 |
| B4218 | B4218-65-2 | Case in chronic stage | 16328154 | 2449.22 | 16230944 | 2142.16 | 87.46 | 97.44 | 93.06 | 56.72 | 7608973 | 140491 |
| B3177 | B3177-62-2 | Case in chronic stage | 16328080 | 2449.21 | 16132876 | 2230.12 | 91.05 | 99.27 | 97.77 | 55.91 | 7596155 | 169668 |
| T1014 | T1014-10-2 | Case in chronic stage | 16328500 | 2449.28 | 16055082 | 2215.51 | 90.46 | 98.88 | 96.81 | 56.91 | 7468117 | 174252 |
| W280 | W280-95-2 | Case in chronic stage | 16079522 | 2411.93 | 15730688 | 2161.77 | 89.63 | 97.83 | 94.85 | 55.73 | 7253442 | 238295 |
| B5869 | B5869-66-2 | Case in chronic stage | 16328056 | 2449.21 | 16189982 | 2161.73 | 88.26 | 98.06 | 94.64 | 56.21 | 7712902 | 74263 |
| B5736 | B5736-68-2 | Case in chronic stage | 16327870 | 2449.18 | 16116136 | 2208.60 | 90.18 | 98.87 | 96.87 | 55.76 | 7534048 | 222367 |
| T1423 | T1423-12-2 | Case in chronic stage | 16328686 | 2449.30 | 15993176 | 2191.92 | 89.49 | 98.95 | 97.03 | 55.97 | 7415304 | 131588 |

| | | | | | | | | | | | | |
|---|---|---|---|---|---|---|---|---|---|---|---|---|
| B9096 | B9096-93-2 | Case in chronic stage | 16328226 | 2449.23 | 16138816 | 2260.88 | 92.31 | 98.41 | 95.53 | 56.77 | 7442342 | 104877 |
| T1215 | T1215-13-2 | Case in chronic stage | 16328962 | 2449.34 | 16019756 | 2210.19 | 90.24 | 98.97 | 97.03 | 55.83 | 7395564 | 123635 |
| B7915 | B7915-70-2 | Case in chronic stage | 16328000 | 2449.20 | 16149846 | 2240.57 | 91.48 | 97.53 | 93.44 | 56.08 | 7489130 | 145845 |
| W247 | W247-96-2 | Case in chronic stage | 16328288 | 2449.24 | 15839782 | 1926.45 | 78.65 | 99.50 | 98.46 | 57.59 | 3526832 | 59957 |
| B9091 | B9091-94-2 | Case in chronic stage | 16328196 | 2449.23 | 16048178 | 2197.00 | 89.70 | 98.13 | 94.89 | 56.11 | 7164396 | 110187 |
| B3690 | B3690-63-2 | Case in chronic stage | 16327726 | 2449.16 | 16130662 | 2220.80 | 90.68 | 98.80 | 96.69 | 56.72 | 7333483 | 235709 |
| B2308 | B2308-61-2 | Case in chronic stage | 16328170 | 2449.23 | 16143650 | 2189.44 | 89.39 | 99.19 | 97.58 | 56.40 | 7563881 | 212609 |
| B7633 | B7633-69-2 | Case in chronic stage | 16327360 | 2449.10 | 15817784 | 2240.88 | 91.50 | 99.11 | 97.38 | 56.13 | 6182402 | 107412 |
| T1444 | T1444-5-3 | Case in recovery stage | 16328594 | 2449.29 | 15962222 | 2162.36 | 88.29 | 98.92 | 96.91 | 55.58 | 7390584 | 274061 |
| T1441 | T1441-7-3 | Case in recovery stage | 16328484 | 2449.27 | 16082708 | 2197.30 | 89.71 | 98.88 | 96.82 | 55.53 | 7654789 | 272701 |
| T1114 | T1114-8-3 | Case in recovery stage | 16329142 | 2449.37 | 16072556 | 2227.94 | 90.96 | 99.00 | 97.19 | 56.22 | 7539747 | 230653 |
| B4255 | B4255-64-3 | Case in recovery stage | 16328154 | 2449.22 | 15973064 | 1985.39 | 81.06 | 97.44 | 93.12 | 56.55 | 6992190 | 41288 |
| T1172 | T1172-19-3 | Case in recovery stage | 16328858 | 2449.33 | 15695696 | 1954.90 | 79.81 | 99.10 | 97.38 | 56.45 | 7442604 | 106010 |
| B5023 | B5023-9-3 | Case in recovery stage | 16328156 | 2449.22 | 16221976 | 2164.27 | 88.37 | 98.01 | 94.54 | 56.00 | 7669027 | 195724 |
| B5462 | B5462-65-3 | Case in recovery stage | 16328252 | 2449.24 | 16202626 | 2114.89 | 86.35 | 98.01 | 94.50 | 56.59 | 7643831 | 196558 |
| B3691 | B3691-62-3 | Case in recovery stage | 16328096 | 2449.21 | 16097394 | 2212.42 | 90.33 | 99.27 | 97.77 | 55.77 | 7443759 | 99960 |

| | | | | | | | | | | | | |
|---|---|---|---|---|---|---|---|---|---|---|---|---|
| T1107 | T1107-10-3 | Case in recovery stage | 16328706 | 2449.31 | 16070058 | 2196.55 | 89.68 | 99.12 | 97.04 | 56.37 | 7623408 | 247676 |
| T1335 | T1335-11-3 | Case in recovery stage | 16328540 | 2449.28 | 15938254 | 2195.02 | 89.62 | 98.93 | 96.94 | 55.88 | 7273395 | 123020 |
| B10639 | B10639-95-3 | Case in recovery stage | 16327160 | 2449.07 | 16118460 | 2208.19 | 90.16 | 99.19 | 97.59 | 55.98 | 7600360 | 325348 |
| B8722 | B8722-66-3 | Case in recovery stage | 16327880 | 2449.18 | 16181900 | 2176.77 | 88.88 | 98.92 | 97.00 | 56.24 | 7681221 | 216705 |
| B7463 | B7463-68-3 | Case in recovery stage | 16328256 | 2449.24 | 16211670 | 2163.89 | 88.35 | 97.75 | 93.93 | 56.00 | 7752545 | 165045 |
| W374 | W374-93-3 | Case in recovery stage | 16051064 | 2407.66 | 15715952 | 2213.51 | 91.94 | 97.86 | 94.77 | 56.72 | 7239140 | 172850 |
| T1241 | T1241-13-3 | Case in recovery stage | 16328844 | 2449.33 | 16166800 | 2200.46 | 89.84 | 98.69 | 96.53 | 56.05 | 7617450 | 143619 |
| B8317 | B8317-70-3 | Case in recovery stage | 16328256 | 2449.24 | 16159196 | 2226.50 | 90.91 | 98.20 | 95.08 | 56.02 | 7506259 | 217378 |
| W458 | W458-96-3 | Case in recovery stage | 16055246 | 2408.29 | 15556674 | 2115.75 | 87.85 | 98.02 | 95.13 | 56.00 | 7109166 | 201213 |
| W298 | W298-94-3 | Case in recovery stage | 16064910 | 2409.74 | 15836608 | 2188.62 | 90.82 | 97.94 | 94.98 | 56.03 | 7429742 | 224653 |
| B7449 | B7449-63-3 | Case in recovery stage | 16327850 | 2449.18 | 16077640 | 2192.37 | 89.51 | 98.82 | 96.76 | 56.82 | 7599391 | 412578 |
| B2337 | B2337-61-3 | Case in recovery stage | 16327710 | 2449.16 | 16056834 | 2211.09 | 90.28 | 98.92 | 96.97 | 56.76 | 6536603 | 141236 |
| B8073 | B8073-69-3 | Case in recovery stage | 16328038 | 2449.21 | 16182888 | 2226.48 | 90.91 | 97.69 | 93.80 | 55.98 | 7633502 | 167426 |
