# Supplementary material for: Dynamic analysis of peripheral blood TCR β-chain CDR3 repertoire in occupational medicamentosa-like dermatitis due to trichloroethylene
Source: Sci Rep. 2021 May 11;11:9971. doi: 10.1038/s41598-021-89431-w (PMC8113444; doi:10.1038/s41598-021-89431-w)
Supplement: Supplementary file 4 — Supplementary Information 4. [file 41598_2021_89431_MOESM4_ESM.pdf]

Article title: Dynamic analysis of peripheral blood TCR β-chain CDR3 repertoire in occupational medicamentosa-like dermatitis due to trichloroethylene

Journal name: Scientific Reports

Author names: Dafeng Lin[1], Dianpeng Wang[1], Peimao Li[1], Xiangli Yang[1], Wei Liu[2], Lu Huang[3], Zhimin Zhang[1], Yanfang Zhang[1], Wen Zhang[1], Naixing Zhang[1], Ming Zhang[1], and Xianqing Huang[1]

Affiliation: [1] Medical Laboratory, Shenzhen Prevention and Treatment Center for Occupational Diseases, Shenzhen 518020, China; [2] Key Laboratory of Modern Toxicology of Shenzhen, Medical Key Laboratory of Guangdong Province, Medical Key Laboratory of Health Toxicology of Shenzhen, Shenzhen Center for Disease Control and Prevention, Shenzhen 518055, China; [3]Fuyong Prevention and Health Care Center, Bao'an District, Shenzhen 518103, China

E-mail address of the corresponding author: david1385@foxmail.com

Supplementary Table S2. Fold changes of the TRBV and TRBV-TRBJ combination frequencies in the OMDT cases relative to the controls from Fig 1 and Fig 2.

Gene segment	*M* (*MAD*)	Fold change relative to controls
TRBV6-4		
Cases in acute stage	0.36 (0.22)	0.38
Cases in chronic stage	0.62 (0.48)	0.65
Cases in recovery stage	0.59 (0.44)	0.62
Controls	0.95 (0.66)	1.00
TRBV7-9		
Cases in acute stage	8.59 (1.26)	1.51
Cases in chronic stage	6.38 (1.43)	1.12
Cases in recovery stage	6.39 (1.27)	1.13
Controls	5.68 (1.58)	1.00
TRBV6-4-TRBJ2-1		
Cases in acute stage	0.05 (0.03)	0.28
Cases in chronic stage	0.08 (0.08)	0.44
Cases in recovery stage	0.08 (0.08)	0.44
Controls	0.18 (0.11)	1.00
TRBV6-4-TRBJ2-2		
Cases in acute stage	0.03 (0.01)	0.27
Cases in chronic stage	0.04 (0.03)	0.36
Cases in recovery stage	0.04 (0.04)	0.36
Controls	0.11 (0.08)	1.00
TRBV6-4-TRBJ2-3		
Cases in acute stage	0.03 (0.02)	0.27
Cases in chronic stage	0.05 (0.04)	0.45
Cases in recovery stage	0.06 (0.05)	0.55
Controls	0.11 (0.08)	1.00

TRBV6-4-TRBJ2-6		
Cases in acute stage	0.004 (0.004)	0.15
Cases in chronic stage	0.007 (0.006)	0.26
Cases in recovery stage	0.016 (0.013)	0.59
Controls	0.027 (0.021)	1.00
TRBV7-9-TRBJ2-1		
Cases in acute stage	1.36 (0.76)	1.97
Cases in chronic stage	0.95 (0.35)	1.38
Cases in recovery stage	0.75 (0.33)	1.09
Controls	0.69 (0.24)	1.00

OMDT, occupational medicamentosa-like dermatitis due to trichloroethylene; M, median; MAD, median absolute deviation
